# Investigating the Defect Structures in Transparent Conducting Oxides Using X-ray and Neutron Scattering Techniques

**DOI:** 10.3390/ma5050818

**Published:** 2012-05-11

**Authors:** Gabriela B. González

**Affiliations:** Department of Physics, DePaul University, 2219 N. Kenmore Avenue, Chicago, IL 60614, USA; E-Mail: ggonza18@depaul.edu; Tel.: +773-325-7398; Fax: +773-325-7334

**Keywords:** transparent conducting oxide, indium-tin oxide, tin oxide, zinc oxide, defect, x-ray diffraction, neutron diffraction, EXAFS, XRF, PDF

## Abstract

Transparent conducting oxide (TCO) materials are implemented into a wide variety of commercial devices because they possess a unique combination of high optical transparency and high electrical conductivity. Created during the processing of the TCOs, defects within the atomic-scale structure are responsible for their desirable optical and electrical properties. Therefore, studying the defect structure is essential to a better understanding of the behavior of transparent conductors. X-ray and neutron scattering techniques are powerful tools to investigate the atomic lattice structural defects in these materials. This review paper presents some of the current developments in the study of structural defects in n-type TCOs using x-ray diffraction (XRD), neutron diffraction, extended x-ray absorption fine structure (EXAFS), pair distribution functions (PDFs), and x-ray fluorescence (XRF).

## 1. Introduction

In most materials, high transparency in the visible region and high electrical conductivity are mutually exclusive properties. However, thin films of transparent conducting oxide (TCO) materials are approximately 90% transparent in the visible region and exhibit electrical conductivity of more than 1000 S/cm [1]. This unique combination of these two properties is very attractive for a wide variety of commercial applications, where TCO materials act as electrodes in optoelectronics devices, such as flat-panel displays and smart windows [2]. Tin oxide was the first commercialized TCO [1]. Currently, commercially-produced TCOs are n-type semiconductors, which include indium oxide, tin oxide, zinc oxide, gallium oxide, and cadmium oxide, that are appropriately doped to improve their electrical behavior while maintaining their transparency. Tin oxide and indium-tin oxide (ITO) are the most widely used TCOs. One advantage of ITO over both pure In_2_O_3_ and SnO_2_ is that ITO films etch more easily and exhibit ideal chemical stability for various device applications. For flat-panel displays the TCO of choice is ITO due to its superior properties and industrial performance.

The transparency of a TCO is due to the wide band gap of the material, which is at least 3.1 eV, corresponding to the highest energy of visible light. The electrical conductivity of a TCO results from the presence of atomic-scale defects, which for n-type semiconductors, bring in donors that increase the electron population. Because the intrinsic population of atomic-scale defects in these oxides is typically very small, the optimization of TCO performance consists of introducing more desirable defects during their synthesis. A post-anneal step in a reducing atmosphere is usually performed to increase the electron population.

The specific types of atomic-scale defects for a given TCO depend on the specific oxide and its synthesis. They may include oxygen vacancies, cation vacancies, oxygen interstitials, cation interstitials, impurity dopants, cation and anion anti-sites, and defect complexes, which consist of a combination of two or more point defects. It is conjectured that more than one type of defect may be present in a material, but the more abundant, non-neutral defects have the greatest effect on the overall conductivity. The charge of these defects is very important, since the overall charge compensation dictates the amount of free donors. In several TCOs with extended dopant concentrations, the dopant population is much larger than the equilibrium limit. High conductivity is expected for these largely doped TCOs, but it is not always observed due to the possible presence of neutral defect complexes which can store the dopants so that they do not contribute to the free electron population [3,4].

It is evident that determining the defect chemistry of TCOs is essential to the understanding of the behavior of these materials and that the optimization of their synthesis would enable an even wider range of commercial applications. Theoretical defect models for TCOs have been developed, but many times the theoretical predictions found in the literature are conflicting [5,6,7,8,9] and lack experimental confirmation. Electrical measurements can provide insight on potential defect models [4]. X-ray and neutron scattering are powerful experimental tools to directly investigate the atomic lattice structural defects in materials. A combination of electrical and scattering measurements can offer the much needed experimental evidence for defect models. This review paper presents some of the current developments in the study of defects in indium-based, tin-based, and zinc-based n-type TCOs using x-ray and neutron scattering techniques.

## 2. Description of Scattering Techniques

### 2.1. X-ray and Neutron Diffraction 

X-ray and neutron diffraction are techniques that give direct information about the structural arrangement of atoms in materials. X-rays interact with the electron cloud surrounding an atom. When the interaction is elastic, the scattered signal contains quantitative information about the location of atoms as well as the type of atoms present. The x-ray scattering factor, *f* (*E*), is expressed as:

(1) $$f\left(E\right)=f_{0}+f\prime \left(E\right)+if\prime \prime (E)$$

where $f_{0}$ is the energy-independent form factor, which is directly related to the number of electrons; f′ and f″ are the anomalous dispersion corrections, which have major contributions only at certain energies.

Because the scattering factor at most x-ray wavelengths is dominated by the $f_{0}$ factor, the x-ray signal is approximately proportional to the number of electrons, and high *Z* elements scatter more strongly than light elements. Therefore, if two elements present in a material are next to each other in the periodic table, their x-ray scattering powers will be nearly indistinguishable at all wavelengths except at the x-ray absorption edges when anomalous scattering effects become important. One example of a TCO where this problem is encountered is indium-tin oxide, where indium and tin have 49 and 50 electrons, respectively, making them almost indistinguishable at most x-ray wavelengths. If a tin atom substitutes for an indium atom on a given site, this substitutional defect could not be quantified with a conventional, lab-based x-ray source. A similar case is antimony-doped tin oxide because antimony has 51 electrons. For these types of materials, an x-ray synchrotron source is necessary so that the experimenter can tune the wavelength to near the absorption edges where the anomalous scattering contributions are enhanced to maximize the contrast between the elements. Alternatively, neutrons instead of x-ray photons can be used to diffract from the samples. 

Neutron diffraction follows the same basic principles as x-ray diffraction (XRD) and the neutron scattering length is equivalent to the x-ray scattering factor. One primary difference is that the neutron scattering length is independent of the number of electrons, since the scattering centers are the neutrons of the atoms. This is especially advantageous in the study of light elements such as oxygen, which suffers from a weak x-ray signal but exhibits a strong neutron signal (scattering length is 5.803(4) fm). For TCOs where oxygen vacancies and oxygen interstitials are important defects, neutron diffraction is a highly valuable technique. Another advantage of neutron diffraction is that most elements with similar number of electrons can be distinguished as in the case of ITO and Sb-doped SnO_2_. The neutron scattering lengths are 4.0652(20)–*i* 9.9539(4) fm for indium, 6.2257(19) fm for tin, and 5.57(3) fm for antimony.

X-ray and neutron diffraction data also contain information about the microstructural defects of crystalline materials. Nanometer-sized grains and lattice strain increase the width of the diffraction peaks. Careful analysis of the peak shape obtained from high-resolution instruments can provide an estimate of the average crystallite size and strain. Crystalline materials produce well defined Bragg peaks that result from the interaction of x-rays with stacks of regular atomic planes. Analysis of the position and intensity of Bragg peaks is used to solve the average, long-range structure of the material. For the case of amorphous materials, where a long-range periodic arrangement of atoms does not exist, the diffraction signal does not contain sharp, strong peaks but rather broad, weak peaks. In those cases, other scattering techniques may be more appropriate to learn about the short-range order of atoms. A brief description of some of those scattering techniques is given below. 

### 2.2. Extended X-ray Absorption Fine Structure (EXAFS) and X-ray Absorption near Edge Spectroscopy (XANES)

EXAFS is an x-ray scattering technique that can provide valuable information about the local structure of both crystalline and amorphous materials. When an x-ray beam traverses a sample of finite thickness, some of the x-ray signal is absorbed by the sample. The absorption coefficient of the sample depends on the energy of the incident x-ray beam and the sample composition. If the energy of the x-ray photon is high enough to excite electrons from inner shells, more electrons become available for photoemission, and the absorption coefficient increases sharply. The energies where discontinuous increases in the absorption coefficient of the sample occur are called absorption edges and are labeled *K*, *L*, *M*, and so on, depending on which shell energy of the absorber electron the edge matches. 

The high-energy sides of an absorption edge have oscillations for up to 2000 eV. This is referred to as the extended x-ray absorption fine structure (EXAFS) region. The photoelectrons that are excited from inner shells interfere with the scattered wave produced by the atoms neighboring the absorbing atom. The interference can be constructive or destructive producing peaks and valleys in the EXAFS region. The EXAFS features therefore contain quantitative information about the neighboring atoms surrounding the absorbing element. In particular, coordination number, distance, and Debye-Waller factors can be obtained. The x-ray absorption near edge spectroscopy (XANES) region includes only about 50 eV immediately after the absorption edge. From this region, chemical information, such as coordination number and valence state of the absorbing element, is obtained. For EXAFS and XANES measurements an x-ray synchrotron source is typically required to scan through the x-ray absorption edge of the element of interest. High statistics, high energy resolution, and a stable monochromatized x-ray beam are essential for successful experiments. 

### 2.3. Atomic Pair Distribution Functions (PDFs)

Atomic PDFs can be obtained for both crystalline and amorphous materials since no long-range order or periodicity is required. Both the Bragg-like peaks and the diffuse scattering component of the x-ray diffraction data can be analyzed. Unlike diffraction, PDFs peaks do not reflect the periodicity found in atomic planes but rather arise from atomic pairs, coordination distances and spheres. A high-energy x-ray or neutron source is typically needed to collect data with a wide range of scattering angles. High-intensity flux and efficient detectors are also required to obtain signals with good statistics. The analysis of the data involves extracting the coherent component of the intensity from experimental patterns using careful corrections. The results consist of radial distributions similar to those obtained from EXAFS, but in this case the information is not element specific. A PDF peak represents a pair of atoms separated by a distance *r*. Similar to the x-ray diffraction experiments where anomalous contributions are enhanced, it is possible to collect data at resonant wavelengths and perform differential analysis to highlight correlations between particular atomic pairs. More detailed explanations on this technique can be found in the literature [10,11,12,13]. 

### 2.4. X-ray Fluorescence (XRF)

As highlighted before, the presence of impurities and dopants in TCOs are important since they can enhance or diminish the electrical properties of the material. The detection and quantification of elements can be obtained via chemical analysis. X-ray fluorescence (XRF) is a non-destructive technique that is commonly used for elemental analysis, and it can detect concentrations as low as a few part per million. A source of energetic photons (x-ray or gamma rays) is required to excite inner electrons from *K* and *L* shells. When electrons from higher shells fill in the inner shells, characteristic secondary radiation is emitted in the form of x-ray photons. This radiation is referred to as x-ray fluorescence. The sample spectrum contains fluorescent lines specific to the elements present in the sample. Analysis of the peak intensities provides quantification of each element. Typical commercial XRF instruments can detect elements as light as magnesium; lighter elements are not easily measured due to attenuation of their signals. This technique, unlike the previous ones, does not provide information about the atomic arrangement of the elements in the sample, but it can be applied to both crystalline and amorphous samples to independently quantify elemental compositions.

## 3. Results on Defects in TCOs

In this manuscript point defects will be expressed using Kröger-Vink notation. The superscripts denote the charge of the defects: a neutral defect is represented by an (^*x*^), a negative defect with ('), and a positive defect with a (^•^) superscript. The subscripts describe the site where the defect is located. For example, $O_{O}^{x}$ represents a neutral oxygen atom on an oxygen site; $V_{O}^{••}$ represents a vacancy on an oxygen site having an effective positive charge of +2; and e′ represents an electron with one negative charge.

### 3.1. Bixbyite In_2_O_3_

**Figure 1:**
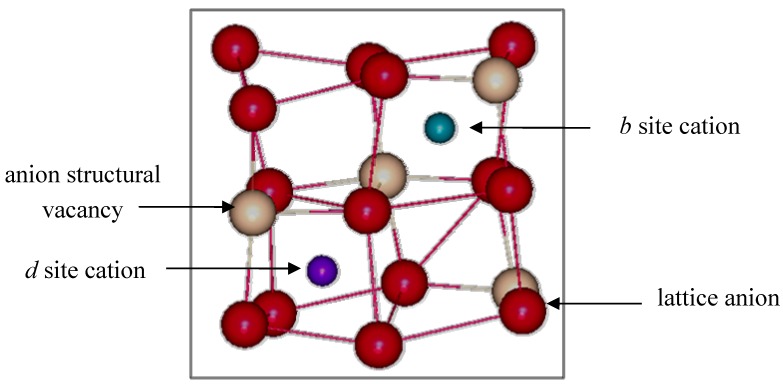
Non-equivalent *b* and *d* cations sites found in bixbyite In_2_O_3_.

The In_2_O_3_ structure was studied using x-ray diffraction on single crystals at two wavelengths [14]. Indium oxide crystallizes in the cubic bixbyite structure, also known as C-type rare-earth sesquioxide with the space group Ia3 (number 206). Its lattice parameter is 10.117(1) Å, and a unit cell has 32 cations and 48 anions. The bixbyite structure is derived from a 2 × 2 × 2 fluorite superstructure that is missing one fourth of the anions, allowing for small rearrangements of the ions. A total of 16 of these “structural oxygen vacancies” are situated at the *c* sites, according to international notation [15]. Depending on the location of these structurally vacant anion sites, two non-equivalent cation sites exist. Figure 1 shows a schematic representation of the two distinct cation sites, which are referred to as equipoints *b* and *d*. 

Eight *b* site cations have six equidistant oxygen anion neighbors at 2.18(1) Å. These oxygen anions lie approximately at the corners of a distorted cube with two anions vacancies along one body diagonal. The remaining 24 cations are found at the more distorted *d* sites, where the two anion vacancies lie along a face diagonal of the distorted cube. There are three cation*_d_* to oxygen bond lengths: 2.13(1) Å, 2.19(1) Å and 2.23(1) Å, which average 2.18(1) Å. The remaining 48 atoms are lattice oxygen anions located at the general position (*e* site) and coordinated to four indium cations. 

The defect structure of pure In_2_O_3_ was indirectly deduced from electrical measurements performed by De Wit *et al.* [16,17,18,19]. They observed a −1/6 dependence of log (*n*) (carrier concentration) as a function of log (*p*O_2_) (partial pressure of oxygen). The non-stoichiometric decomposition and its corresponding equilibrium constant are described below:

(2) $$2In_{In}^{x}+3O_{O}^{x}⟺2In_{In}^{x}+3V_{O}^{••}+6e^{\prime }+\frac{3}{2}O_{2}$$

(3) Kvac=[VO••]3n6PO232

The electroneutrality condition is

(4) $$n=2[V_{O}^{••}]$$

Substituting Equation (4) into Equation (3), the carrier concentration dependence on *p*O_2_ is

(5) n∝PO2−16

Therefore, the observed −1/6 slope indicated that undoped In_2_O_3_ exhibits n-type conductivity due to the presence of oxygen vacancies under reducing conditions. These vacancies would be formed by removing some of the lattice anions located at the *e* sites and therefore introducing non-stoichiometry in the sample to obtain In_2_O_3–*x*_ where *x* depends on the oxidizing conditions of the material, but it is usually less than 0.01 [16]. Due to the small population of oxygen vacancies that is predicted in pure indium oxide (less than 0.33% of the lattice oxygen site), no x-ray or neutron diffraction experiment has been able to confirm their existence. XRF techniques cannot determine the In/O ratio with such high precision either. However, theoretical models agree that the prevailing defects causing n-type conductivity in pure indium oxide are oxygen vacancies [5].

### 3.2. Bixbyite ITO

Indium-tin oxide is obtained by doping In_2_O_3_ with tin. The bixbyite structure of the parent indium oxide is kept up to the maximum solubility limit, and for temperatures lower than 1345 °C in samples at thermodynamic equilibrium [20,21]. Frank and Köstlin [4] pioneered the study of defect models for ITO. They proposed different clusters of defects to fit observed electrical properties. A summary of their model is described in the following section.

#### 3.2.1. Frank and Köstlin Model for ITO

Resistivity, carrier concentration, and mobility were measured at room temperature as a function of Sn-doping and *p*O_2 _on ITO thin films. Under reducing conditions, Frank and Köstlin observed that for all doping levels, a −1/8 slope on log (*n*) *vs.* log (*p*O_2_) was followed by a plateau. The transition at which the carrier concentration plateaued depended on the Sn content. The carrier content increased with tin doping level up to a certain Sn concentration (~5 cation%, depending on *p*O_2_), and then decreased. On the increasing part of the curve, the slopes became increasingly shallow with increasing Sn doping, *i.e.*, the more Sn, the less efficiently the electron population increased. 

At doping levels <1 cation% and very high reducing conditions (~10^−20^ bar), the observed carrier concentration was proportional to the tin doping level *C_Sn_*. Such a behavior would indicate that every Sn**^•^** donor contributes one electron when the material is highly reduced. However for tin doping levels higher than 1 cation%, regardless of *p*O_2_, the carrier concentration to tin concentration dependence deviated to slopes smaller than 1. In order to fit their observations, they postulated the existence of various Sn-O*_i_* clusters.

The −1/8 slope of log (*n*) *vs*. log (*p*O_2_) was explained from the disassociation of reducible (2Sn**^•^**O*_i_*″)*^x^* clusters. According to this model, the two tin ions are not nearest neighbors, and when the partial pressure of oxygen decreases it is possible to ionize this cluster, as shown in Equation (6).

(6) $${\left(2Sn^{•}O_{i}\prime \prime \right)}^{x}↔2Sn^{•}+2e^{\prime }+\frac{1}{2}O_{2}(g)$$

(7) K=[Sn•]2n2PO212[(2Sn•Oi″)x]

Assuming that oxygen vacancies are minority species, the electron concentration is equal to the concentration of Sn**•**. Therefore, the carrier concentration dependence agrees with experimental observations. When the Sn doping level is small, n∝CSn14 and the total Sn concentration in the sample can be written as

(8) $$C_{sn}=\left[Sn^{•}\right]+2[{\left(2Sn^{•}O_{i}\prime \prime \right)}^{x}]$$

The n∝CSn14 dependence agrees with the experimental observations at oxidizing conditions (<10^‒2^ bar) and at low tin doping levels (<4 cation%). However, the presence of both reducible clusters and Sn• species explained only some of the observations. For very highly reducing conditions and doping levels greater than 1 cation%, the observed carrier concentration vs. doping concentration exhibited slopes smaller than 1, and the slopes decreased with increasing *p*O_2_. Frank and Köstlin proposed the existence of an “irreducible” ${\left(2Sn^{•}O_{i}\prime \prime 3O_{O}\right)}^{x}$ cluster. The cluster also has 2 non-nearest tin neighbors that tightly bind to one oxygen interstitial and to three nearest lattice oxygens.

At high Sn concentrations (>5 cation%), adding more tin decreased, rather than increased, the electron population. Therefore, Frank and Köstlin hypothesized the presence of another cluster formed by the aggregation of a reducible and an irreducible cluster.

(9) $${\left(2Sn^{•}O_{i}\prime \prime \right)}^{x}+{\left(2Sn^{•}O_{i}\prime \prime 3O_{O}\right)}^{x}↔({\left(2Sn^{•}O_{i}\prime \prime \right)}^{x}{\left(2Sn^{•}O_{i}\prime \prime 3O_{O}\right)}^{x})$$

The total Sn concentration is then expressed as

(10) $$C_{Sn}=\left[Sn^{•}\right]+2[{\left(2Sn^{•}O_{i}\prime \prime \right)}^{x}]+2[{\left(2Sn^{•}O_{i}\prime \prime 3O_{O}\right)}^{x}]+4[\left({\left(2Sn^{•}O_{i}\prime \prime \right)}^{x}{\left(2Sn^{•}O_{i}\prime \prime 3O_{O}\right)}^{x}\right)]$$

Equation (10) has a mixture of effective and non-effective tins. The non-effective species act as a sink of electrons, while the effective tins actually donate electrons. The total effective Sn concentration is defined as:

(11) $$C_{Sn_{e}}=\left[Sn^{•}\right]+2[{\left(2Sn^{•}O_{i}\prime \prime \right)}^{x}]+2[\left({\left(2Sn^{•}O_{i}\prime \prime \right)}^{x}{\left(2Sn^{•}O_{i}\prime \prime 3O_{O}\right)}^{x}\right)]$$

#### 3.2.2. Defect Structure Study of ITO Using X-ray and Neutron Techniques

A comprehensive study on the defect structure of indium-tin oxide (ITO) was performed by the author [22,23,24]. Powder samples of three tin concentrations (0%, 3%, and 9% Sn) were prepared under oxidizing and reducing conditions. Electrical conductivity and thermopower were measured and the samples were also characterized using EXAFS, anomalous x-ray diffraction, neutron diffraction, and XRF techniques. 

Undoped In_2_O_3_ and 3% doped ITO bulk samples were sintered at 1350 °C in air. The samples were subsequently annealed in air at 800 °C for 15 h and quenched to room temperature. An extra annealing step was performed for some samples, either at 800 °C in CO/CO_2_ (*p*O_2_ ~ 10^−14^ atm) for 65 h or at 500 °C in forming gas (4% H_2_/96% N_2_) for 6 h. Nano-ITO powders with 9% Sn were sintered at 700 °C for 1 h. The nano samples were then annealed in air for 5 h at 500 °C and quenched to room temperature. Some of these samples were also reduced in forming gas at 500 °C for 6 h and quenched to room temperature.

*In situ* conductivity and thermopower measurements were performed at 800 °C for the ITO bulk samples and at 500 °C for the nano-ITO samples. In agreement with Frank and Köstlin [4], a −1/8 slope of log (conductivity) *vs.* log (*p*O_2_) was followed by a plateau at highly reducing conditions. Furthermore, a 1/24 slope of normalized thermopower *vs.* log (*p*O_2_) was also followed by a plateau. The observed *p*O_2_ dependences of conductivity and thermopower support the ionization of reducible clusters, while the plateau regime is consistent with the existence of irreducible clusters.

EXAFS measurements were performed at the 5-BMD beamlime of the Advanced Photon Source (APS). The experiments were performed in transmission mode at the In *K* absorption edge (27,940 eV) and at the Sn *K* absorption edge (29,200 eV). The measured signal ranges for the In edge were from *k* = 3.3 to 14 Å^−1^ and from 3.3 to 12.6 Å^−1^ at the Sn edge. The indium to oxygen distances in bulk and nano-ITO samples were 2.18 to 2.19 Å, which are similar to the In-O distance in undoped In_2_O_3_ (2.18 Å). The first coordination shell consisted of approximately 6 neighbors in all samples. The tin to oxygen distances ranged from 2.05 to 2.08(1) Å in ITO samples, which was shorter by approximately 0.12 Å compared to In-O. This result agreed with EXAFS experiments performed by other research groups [25,26,27] and was expected based on the 0.10 Å radial difference between Sn^4+^ and In^3+^ ions. As a comparison, in SnO_2_, the Sn-O distance is 2.045 Å. The first Sn-O coordination shell in ITO consisted of approximately 6 neighbors; however, due to the 10% uncertainty in this number, it was difficult to accurately distinguish a coordination number of 6 from 7. However, the EXAFS result definitely suggested that oxygen anions moved closer to tin than to indium in all of the ITO samples. 

Anomalous x-ray diffraction experiments were performed at the APS (5-BMC beamline). High-resolution, x-ray diffraction patterns on all quenched samples were measured at room temperature below and at the In *K* absorption edge to maximize the contrast between indium and tin. Time-of-flight neutron powder diffraction patterns were also measured at room temperature, using the Special Environment Powder Diffractometer, located at (alas, shuttered) the Intense Pulsed Neutron Source at ANL. The measured *d* ranges for the x-ray data were 0.87 to 4.13 Å and 0.50 to 3.00 Å for the neutron data. Both x-ray and neutron data sets were refined simultaneously using the Rietveld method [28].

The overall tin concentration in the samples was determined by XRF and confirmed by Rietveld analysis. The nano-powders were phase-pure, while the bulk samples had 96.5 wt % ITO and 3.5 wt % In_4_Sn_3_O_12_. The nano-powders had approximately 9% Sn, which was beyond the solubility limit. This extended solubility was attributed to the metastability of nano-particles, where more tin can be incorporated in the structure. The combined analysis of diffraction patterns showed that in ITO tin substitutes into both *b* and *d* cation sites but with a preference of the *b* cation site, which has higher symmetry (see Figure 1). This result was in agreement with Mössbauer experiments [27]. The percentage of tin occupation in the *b* sites for bulk-ITO ranged from 5(3) to 9(3)%, while for the *d* site it was 1(1) to 2(1)%. For the nano-ITO samples, the tin occupancies were 15(4) to 25(5)% and 3(1) to 7(1)% for the *b* and *d* sites, respectively. 

All ITO samples exhibited the presence of oxygen interstitial atoms located at the *c* sites, previously referred to as the anion structural vacancy site in bixbyite. On the other hand, for the undoped In_2_O_3_ samples the *c* site remained vacant. The amount of O*_i_* present varied from sample to sample, but consistently the oxidized samples had higher O*_i_* populations than the reduced samples. For bulk-ITO, the O*_i_* occupational percentages in the samples were: 2.6(5)% when oxidized; 2.1(5)% after forming gas reduction; and 1.0(5)% after CO/CO_2_ reduction. For the nano-ITO samples, the O*_i_* populations were higher: 10.4(8)% and 5.7(7)% under oxidizing and reducing conditions, respectively. In a 2 × 2 × 2 fluorite superstructure the equivalent position of the *c* site would be *x* = *y* = *z* = 0.125 fractional lattice units. The refined positions of the O*_i_* site ranged from 0.80(5) to 0.086(3) for bulk-ITO and from 0.085(2) to 0.091(1) for nano-ITO. The O*_i_* position in reduced samples was consistently smaller for both bulk- and nano-ITO compared to the oxidized specimens.

The ratio of total Sn to O*_i_* was calculated for all samples and compared to the value of 2 proposed by Frank and Köstlin for both reducible and irreducible clusters. For bulk oxidized ITO, the Sn/O*_i_* ratio was 2.2(4); and upon reduction in forming gas, it increased to 2.7(6). Because the CO/CO_2_ treatment dramatically decreased the O*_i_* population, the ratio for this sample was 5.5(2.4). For nano ITO, the Sn/O*_i_* ratio was 1.7(1) and upon reduction it became 3.1(3). The close proximity of the experimental values in oxidized ITO to the ratio of 2 supported the idea that the substitution of two Sn^•^ positively charged defects in the In_2_O_3_ lattice bring in an additional O*_i_*" species to charge compensate, as in the reducible (2Sn^•^O*_i_*″)*^x^* cluster. If all the Sn and O*_i_* species were in reducible clusters, upon reduction all those complexes would be ionized resulting in an increase in conductivity and in the total removal of O*_i_* defects. However, since the highly reduced sample contained some O*_i_* population and a plateau in conductivity, the presence of irreducible clusters was also supported.

**Figure 2:**
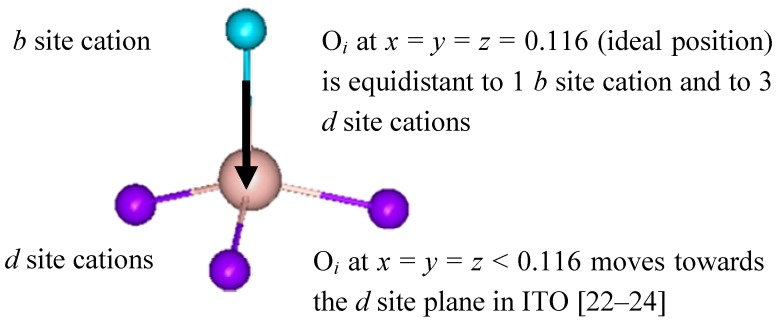
Nearest cations surrounding an oxygen interstitial located at the *c* sites in the bixbyite structure.

Theoretical models [3,29,30] propose that the non-reducibility in ITO is due to the aggregation and proximity of tin cations around oxygen interstitials. As mentioned earlier, the refined O*_i_* positions (0.80 to 0.91) were smaller than in the fluorite structure (0.125). In bixbyite, the ideal position would correspond to 0.116 atomic lattice coordinates, such that an oxygen interstitial is located 2.35 Å from 1*b* and 3*d* cations, as shown in Figure 2. The smaller experimental coordinates corresponded to a displacement of the O*_i_* species towards the *d* site plane and away from the *b* cation. The distance from O*_i_* to *d* sites was 2.2(1) Å, while the distance to the *b* cation increased to 2.8(1) Å. Theoretical calculations showed that isolated O*_i_^x^* defects would be located at 0.110 fractional coordinates, which is far from the experimental value. However, when clustering of tin and oxygen interstitials was modeled, the agreement with experiments dramatically improved. These theoretical models also predicted that tin strongly preferred the *d* site in clusters where only nearest neighbor O*_i_* species participate. When looking at second and higher coordination shells, then tin preferentially substituted into the *b* site, in agreement with the experimental observations. 

Other theoretical studies have tried to explain the *b* site preference that was experimentally observed by proposing that the synthesis conditions may shift the tin distribution in the indium sites [31]. Further theoretical investigations have corroborated that the nonreducibility of defects was due to the aggregation of tin cations around oxygen interstitials, but that also the chemical potential of conduction electrons increased with decreasing O*_i_* population, thus limiting the maximum concentration of electrons available for conduction [32]. 

Rietveld refinements also revealed that the bixbyite lattice parameter expanded with increasing tin concentration. Considering that the radius of Sn^4+^ is 0.1 Å smaller than In^3+^, cell contraction was expected with tin substitution. Similar increases in the ITO lattice parameter have been reported in the literature [4,21,27], and attributed to an incomplete charge compensation of Sn**^•^** defects by O*_i_*″ defects. As more positively charged tin defects are created, the repulsion among these species increases resulting in cell expansion. This explanation was consistent with the further expansion that was observed in reduced samples, where less O*_i_*″ species were present enhancing the repulsion. 

### 3.3. Zinc and Tin Doped Indium Oxide (ZITO)

Another indium-based transparent conducting oxide results from co-doping indium oxide with zinc and tin. The material is referred to as ZITO and is a relatively new TCO material. It is an attractive substitute to ITO since zinc and tin replace indium, which has become more expensive and scarce. Since the first literature report of ZITO in 1995 [33] many studies on the properties, range of compositions, and defects have been published [34,35,36,37,38,39,40,41,42,43,44,45,46,47,48,49]. N-type bixbyite ZITO materials having more tin than zinc exhibit electrical properties comparable to ITO. Recently, ZITO’s defect structure has been studied using crystalline powders [43,44], amorphous powders [46], and amorphous thin films [47,48]. Crystalline ZITO can exhibit the bixbyite or the corundum phase. For the corundum phase, up to 70% co-substitution is possible [44], while according to Palmer *et al.* [35] up to 40% of the indium can be replaced with Sn^4+^ and Zn^2+^ in bixbyite ZITO. The preparation of the corundum phase requires a more complicated process since high pressure (~70 kbar) and high temperature (1000 °C) are required to transform the material [44].

#### 3.3.1. EXAFS Studies of Crystalline ZITO (c-ZITO) with the Bixbyite Structure

##### 3.3.1.1. Study on Bixbyite c-ZITO Powder Materials

The local structure of bixbyite ZITO was investigated by Hoel *et al.* [43] on powders using EXAFS. The materials studied were In_2–2*x*_Zn*_x_*Sn*_x_*O_3_ (*x* = 0.1, 0.2, 0.3, and 0.4) and were referred to as ZITO-10, ZITO-20, ZITO-30, and ZITO-40. The measurements were performed at the APS in transmission mode at the In, Sn, and Zn *K* absorption edges. Because EXAFS experiments do not have enough resolution to distinguish the *b* and *d* cation site environments, the results provided an average of distances for each coordination shell. Therefore, it was not evident whether the dopants substituted for In randomly or if there was a site preference as determined in the ITO defect structure study [22,23,24]. Hoel *et al.* [43] assumed that tin and zinc substituted randomly in the indium sites. Laboratory XRD measurements confirmed the phase purity of the samples and indicated that the In_2_O_3_ bixbyite unit cell contracted linearly as tin and zinc co-substitutions increased, consistent with the smaller radii of the dopants.

Similar EXAFS features were observed for ZITO and In_2_O_3_ bixbyite powders at the In edge. The main difference was the decrease of the amplitudes for both the In-O and In-In/Sn/Zn bonds in ZITO as the parameter *x* increased. This decrease was attributed to the weaker scattering of Zn which has 30 electrons compared to In and Sn, which have 49 and 50 electrons, respectively. Furthermore, Zn interfered destructively with the In and Sn EXAFS signals due to a phase shift resulting in an overall weaker signal. The coordination number for all 4 measured In shells was fixed to 6. The In-O distance for the first shell averaged 2.161(4) Å for all ZITO compositions, compared to 2.18 Å in In_2_O_3_. The next three shells corresponded to In-In/Sn/Zn. For these cation shells higher co-substitution consistently resulted in shorter bond lengths. For the second shell, the distances ranged from 3.348(5) Å for ZITO-10 to 3.321(7) Å for ZITO-40. For the third shell and fourth shell, the bond lengths ranged from 3.820(5) Å to 3.789(8) Å, and from 5.068(7) Å to 5.027(10) Å, respectively. These observations were consistent with the unit cell contraction measured from XRD and with the smaller radii of Zn^2+^ and Sn^4+^ compared to In^3+^.

EXAFS data for ZITO-20, ZITO-30 and ZITO-40 were reported at the Sn edge. EXAFS results indicated that the tin local environment for bixbyite ZITO was similar to that of In in the bixbyite structure. The same structural features were observed, however, the amplitude of the ZITO spectra again decreased with increasing *x*, due to destructive interference from Zn. The first four coordination shells were fixed to 6 neighbors. The Sn-O first shell distance contracted owing to the smaller Sn^4+^ radius, and it was actually closer to the bond in SnO_2_ (2.05 Å) than to the bond in In_2_O_3_ (2.18 Å) or in ZITO at the In edge (2.16 Å). The Sn-cation shells were also shorter than the In-cation shells. However, unlike the In shells, they showed a very modest bond-length shortening with increasing co-substitution, and the actual distance ranges were almost independent of composition within experimental uncertainties. The range of distances were 2.080(7) Å for ZITO-20 to 2.073(6) Å for ZITO-40 for the first shell, 3.36(1) Å to 3.34(1) Å for the second shell, 3.83(1) Å to 3.81(1) Å for the third shell, and 5.02(1) Å to 4.99(1) Å for the fourth shell. 

The coordination type in zinc was determined using XANES measurements at the Zn edge. The Zn-O shell in ZnO is tetrahedral, and as described before, in bixbyite the In-O shell is octahedral. XANES experimental results indicated that the local environment was octahedral as in bixybite In_2_O_3_, so the first three zinc coordination shells were fixed to 6 neighbors. As in the Sn edge, data for ZITO-20, ZITO-30 and ZITO-40 were reported. The Zn-O first shell distance also contracted owing to the smaller Zn^2+^ radius, and within experimental uncertainty, it was the same as the Sn-O shell. The range of distances were 2.084(7) Å for ZITO-20 to 2.075(6) Å for ZITO-40 for this first shell. Unlike the In results, higher coordination shells did not seem to shorten with increasing co-doping taking into account the larger experimental uncertainties at the Zn edge. The average distances were 3.30(1) Å and 3.74(2) Å for the second shell and third shells, respectively. The higher uncertainties were attributed to a more disordered local environment.

##### 3.3.1.2. Study on c-ZITO Thin Films

Proffit *et al.* [47,48] studied the local structure of a bixbyite ZITO thin film deposited on a sapphire substrate. The sample had an In-Sn-Zn composition of 78-12-10 as determined using XRF in a scanning electron microscope. The conductivity was 1565 S/cm; the Hall mobility was 41 cm^2^/Vs; and the carrier concentration was 2.4 × 10^20^ electrons/cm^3^. EXAFS and XANES measurements were performed at the In, Zn and Sn *K*-edges using a Ge solid-state detector in fluorescence mode using three different orientations. The incident angles were 6°, 30° and 50° with respect to the sample surface. The data were collected at the 5-BMD beamline of the APS. The EXAFS data for *k* = 3.2 to 10 Å^−1^ and *R* = 1.0 to 4.1 Å were fit.

XANES spectra indicated that the oxidation states of the cations were Zn^2+^, In^3+^, and Sn^4+^. Furthermore, all the cations exhibited octahedral coordination, similar to the results in crystalline bixbyite ZITO [43]. The In-O bond length was 2.17(3) Å with 5 to 6 neighbors depending on the incident angle. The Sn-O bond was split 2.08/2.25(3) Å with a total of approximately 6 neighbors. The Zn-O bond was also split 2.04/2.18(3) Å with a total of approximately 5 neighbors. 

The cation-oxygen average bond lengths were similar to the In-O bond in bixybite (2.18 Å). The uncertainty in the coordination numbers was conservatively on the order of 10%, so taking into account these uncertainties it was difficult to distinguish 5 from 6 neighbors. However, because for all 3 orientations of the films the results consistently showed that the zinc cation was under-coordinated, the authors postulated a redistribution of oxygen near Zn. One lattice anion displaced to a *c* site near In and Sn can leave behind an oxygen vacancy. The presence of an additional oxygen vacancy around Zn would result in 5 neighbors instead of 6. While the presence of an extra oxygen around In and Sn would increase the coordination number of these cations to 7, the overall stoichiometry and charge balance would remain the same. However the local environment around each of the cations would be different. Measurements on ZITO samples by Harvey *et al.* [42] found the same −1/8 slope of log (conductivity) *vs.* log (*p*O_2_) that was reported before for ITO materials [4,22,23,24]. The defect mechanism responsible for the n-type behavior in ZITO therefore has been attributed to Frank and Köstin defects similar to those in ITO [40]. Samples with excess Sn (~2.5%) result in increased conductivities compared to samples with excess Zn (~2.5%) or with nominal [Sn] = [Zn] compositions. The results therefore indicated that ZITO samples with excess aliovalent Sn (Sn > Zn) exhibited donor populations when reduced, as in the case of ITO. Assuming that the Zn dopants were completely compensated by isovalent Sn species, Proffit [48] calculated that the remaining 2% of Sn would result in conductivities comparable to those measured. The zinc cations therefore would not contribute to the conductivity but would allow for higher levels of indium substitution in ZITO compared to ITO. 

#### 3.3.2. Defect Structure Study of Amorphous ZITO (a-ZITO) Using X-ray Techniques

Amorphous ZITO samples are attractive due to their high electrical mobilities compared to crystalline materials. The lack of grain boundaries reduces the scattering of charge carriers. When amorphous ZITO samples are heated, the electrical conductivity increases and reaches a maximum just before crystallization occurs. The carrier concentration seems to remain unaffected, so the increase in conductivity has been attributed to the mobility [49]. In amorphous samples, the definition and the role of defects is less apparent than in crystalline samples. It is clear that unlike crystalline materials, amorphous samples lack long-range order; however, they seem to exhibit short-range order as reported by studies on amorphous ZITO powders and thin films, which are described next. 

##### 3.3.2.1. Study on a-ZITO Powder Materials

A comprehensive investigation of the defect structure in a-ZITO powders was performed by Hoel *et al.* [46], and a summary of their results is described here. The samples were In_2−2*x*_Zn*_x_*Sn*_x_*O_3_ where *x* = 0.2, 0.3, and 0.4. The powders were synthesized using a precipitation and calcination process. The characterization techniques included XRD, EXAFS, XANES, total scattering PDF, and transmission electron microscopy (TEM). The authors compared the results of the a-ZITO with those of crystalline bixbyite and corundum In_2_O_3_. TEM images revealed that 20 to 50 nm particles aggregated in a random manner. Diffraction data were consistent with an amorphous arrangement of atoms.

High resolution XRD experiments were performed at room temperature at the 11-BMC beamline of the APS. The diffraction pattern consisted of broad peaks whose positions corresponded to approximately the *d*-spacings of crystalline bixbyite and corundum. *In situ*, high temperature, XRD measurements, were performed at the 5-BMD beamline of the APS using a monochromatized beam of 0.654 Å. Data on the precursors were collected from room temperature up to 568 °C in transmission mode using a 2D detector to study the crystallization process. At room temperature only the crystalline precursor diffraction peaks were present. At 300 °C the signal became completely amorphous. As the sample was heated to even higher temperatures, the diffraction peaks gradually sharpened. After heating to ~560 °C, the sample crystallized into a mixture of bixbyite and corundum phases. 

EXAFS data were measured at the In, Sn, and Zn *K* edges in transmission mode at the APS. The amount of zinc and tin did not have a significant effect on the EXAFS results at the In, Sn and Zn edges. Several constraints were applied to the data at the In edge: only In neighbors were considered in the fits; the In-O shell coordination number was set to 6 as in bixyite and corundum In_2_O_3_; the coordination number for edge- and corner-sharing cations were set to be an average of 6 for each case. The In-O distance was 2.140(4) Å, slightly shorter than in bixbyite ZITO (2.16 Å). The second coordination shell was split and consisted of 3.2(4) edge-sharing indium neighbors at 3.28(1) Å, and 2.8(4) neighbors at 3.41(2) Å. The third shell was also split and consisted of 3.2(4) corner-sharing indium neighbors at 3.64(1) Å and 2.8(4) neighbors at 3.88(2) Å. The tin and indium local environments exhibited structural differences. Constraints were also applied at the Sn edge: only indium neighbors were fit for distances ranging from 3 to 4 Å; and the Sn-O coordination number was set to 6 as in rutile SnO_2_. The Sn-O distance was 2.058(3) Å. The second, third and fourth shells consisted of Sn-In neighbors and were as follows: 4.1(4) neighbors at a distance of 3.27 Å; 3.0(2) cations located 3.48(1) Å apart; and 3.9(4) neighbors at 3.72(1) Å. For the Zn edge, XANES spectra were analyzed to determine if the coordination was either octahedral as in bixbyite and corundum ZITO or tetrahedral as in ZnO. The results indicated that for a-ZITO the Zn-O were tetrahedrally coordinated. EXAFS experiments showed that the Zn-O shell was 1.97(2) Å when the number of neighbors was fixed to 4 in a-ZITO. This distance agreed with tetrahedral coordination. No shells beyond 2 Å were observed; this behavior differed from that of the crystalline bixbyite and corundum phases.

PDFs measurements were collected in transmission mode at 6-ID-D of the APS at ANL using 87.06 keV x-rays and a 2D detector. Relatively sharp peaks corresponding to atomic pairs of less than 5 Å were obtained for all compositions. The amount of zinc and tin did not seem to have a significant effect on the PDF results, consistent with the EXAFS observations. The first PDF peak at 2.14 Å corresponded to an average of In-O, Sn-O, and Zn-O pairs. The second and third peaks had similar integrated intensities and occurred at 3.29 Å and 3.73 Å corresponding to edge and corner-sharing cation-cation pairs, respectively. At distances higher than 5 Å, the peaks for a-ZITO became broader indicating a decrease in order. For comparison purposes, the authors measured PDFs of bixbyite and corundum In_2_O_3_ and found that those crystalline samples exhibited sharp peaks up to 20 Å (the maximum range of measured results). 

From the above experiments the authors concluded that a-ZITO had structural differences with bixbyite and corundum ZITO. In the crystalline phases, all the cations are octahedrally coordinated to oxygen; however the Zn environment exhibited tetrahedral coordination in the amorphous phase, which probably prevented crystallization of the samples. The In-In and Sn-In local environments differed from each other, while the Zn-In environment seemed to be too disordered to exhibit any measurable structure. At 568 °C, a-ZITO crystallized into a mixture of corundum and bixbyite phases. At 700 °C, both crystalline phases still coexisted; however, higher zinc and tin substitutions favored the presence of corundum.

##### 3.3.2.2. Study on a-ZITO Thin Films

Proffit [48] studied the local structure of several amorphous ZITO thin films. A set of samples with a Sn/Zn ratio of 12/14 (actual ratio 11.9/13.5, also referred to as ZITO30, since approximately 30% of indium is replaced with Sn and Zn) was prepared to study the effect of *p*O_2_ during growth. The *p*O_2_ values were 0.5 mTorr, 7.5 mTorr, and 15 mTorr. The corresponding conductivities were 7, 1457, and 342 S/cm; the Hall mobility values were 1.4, 49.5 and 53.0 cm^2^/Vs, while the electron concentrations were 2.9 × 10^20^, 1.7 × 10^20^, and 3.7 × 10^19^ cm^−3^. Since the 7.5 mTorr conditions resulted in the best electrical properties, two other compositions were prepared at this *p*O_2_: 16/14 (actual ratio 15.5/13.6) and 18/13 (actual ratio 17.8/13.3). The corresponding conductivities for these samples were 1226 and 1431 S/cm, while the Hall mobilities were 48.4 and 46.7 cm^2^/Vs, and the electron concentrations were 1.6 × 10^20^ and 1.9 × 10^20^ cm^−3^. The electrical properties seemed to be optimized for medium *p*O_2_ environments (7.5 mTorr). However, the Sn/Zn ratio did not have an effect on the electrical performance of the amorphous samples as it did in the bulk crystalline samples, where higher In compositions resulted in increased conductivities for Sn-rich samples (~2.5%) [40]. It should also be noted that the 12/14 Sn/Zn composition film had an excess of zinc, but it resulted in n-type conductivity with high carrier concentration and mobility, unlike bulk crystalline samples, where Zn-rich samples exhibited n-type behavior with inferior electrical properties. Another sample having a total of 70% of indium replaced with zinc and tin was prepared at 7.5 mTorr. This composition in crystalline samples was well beyond the solubility limit, which is about 40%. 

As in the crystalline thin film sample of composition 12/14, XANES measurements for most samples, indicated that the cations present in a-ZITO were Zn^2+^, In^3+^ and Sn^4+^. The only sample where Sn^2+^ might have been present was the sample prepared at 0.5 mTorr. The In and Sn XANES signals were very similar for the amorphous and crystalline samples. However, the Zn signal was broader due to a higher degree of disorder. The radial distribution functions showed that only first shell ordering can be measured in amorphous samples. The In-O bond length was 2.14(2) Å with a coordination number of 5.0(4). The bond length was similar to those of the crystalline film and powders within experimental uncertainty. The Sn-O bond length was 2.07(3) Å, which matched the shorter distance in the crystalline film. This distance was also consistent with the amorphous powders and with the length in SnO_2_. The coordination number in a-ZITO films was 5.9(3), except for the sample prepared at 0.5 mTorr, whose coordination number was 4.1(3). The Zn-O bond length was even shorter, 1.98(1) Å, also comparable to the a-ZITO powders and to the length in ZnO. The number of oxygen neighbors in the a-ZITO films was 3.3(2), which is much smaller than in the c-ZITO thin film.

Anomalous PDF measurements on a modest data range up to *q* = 9 Å^−1^ were performed at the Zn *K*-edge on a film similar to the 12/14 composition. While the uncertainties of these results were large due to the limited measured range, the PDF observations were consistent with the EXAFS results for the Zn local environment. Similar trends in the coordination numbers for the first shell in c-ZITO and a-ZITO films were observed, with Zn having fewer neighbors than In and Sn. The role of Zn in a-ZITO was less evident compared to c-ZITO where it served to compensate some of the Sn species and allowed for higher doping levels. In amorphous samples, the definition and the role of defects is less apparent than in crystalline samples. In the case of ZITO the Sn/In ratio had no effect on the electrical properties, while in crystalline samples the composition had a major effect attributed to different populations of defects. ZITO samples crystallized at higher temperatures (300–345 °C [48]) compared to ITO (185–230 °C [50]) and In_2_O_3_ (165–210 °C [50,51]), therefore zinc may play a key role in increasing the stable temperature range for a-ZITO.

As mentioned before, crystallization of amorphous samples typically causes a reduction in the mobility of the samples, due to the increased scattering of carriers by grain boundaries. Buchholz *et al*. [49] reported that the conductivity of amorphous samples increased with temperature and then decreased when crystallization occurs. The authors attributed the enhanced conductivity during annealing to the homogenization of defect populations and to the strain relaxation in the sample. Proffit [48] studied the crystallization of ZITO films using *in situ* glancing incidence x-ray diffraction (GIXRD). Consistently, the addition of zinc to the sample resulted in higher crystallization temperatures. Samples with composition 12/14 (ZITO30) that were deposited at room temperature and 5 mTorr O_2_ crystallized between 300 and 345 °C. In another experiment, the effect of substrate temperature during deposition at 5 mTorr was also explored on ZITO samples with 30%, 50% and 70% co-doping levels. The temperatures measured were 25 °C, 200 °C and 400 °C. For ZITO30 crystallization started at a substrate temperature of 200 °C, while for ZITO50, the temperature was 400 °C. The ZITO70 sample remained amorphous at 400 °C, however the substrate temperature was not increased further to prevent the evaporation of zinc. The optical electrical properties of ZITO50 were studied from −100 °C up to 400 °C, and it was determined that the optimal temperature for optical performance was 25 °C. However for this sample and at the experimental temperatures studied, at 200 °C the amorphous sample exhibited the best electrical properties. For ZITO30, the best electrical properties were at 25 °C, when again the sample was still in the amorphous phase. The ZITO30 sample was more conductive than the ZITO50 sample due to smaller carrier concentrations. These results suggested that composition, substrate temperature and further annealing modify the electrical and optical properties of a-ZITO materials, so it is possible to tune and optimize these properties to satisfy the demands for a range of applications.

### 3.4. Rutile Tin Oxide (SnO_2_) 

**Figure 3:**
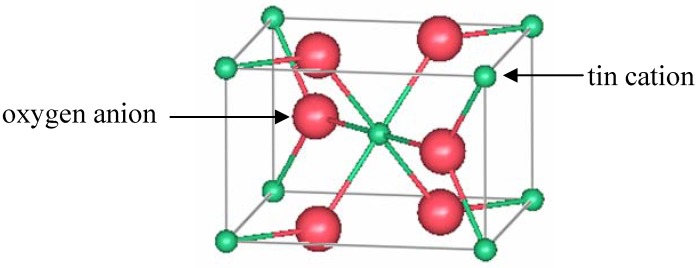
One unit cell of rutile SnO_2_.

Tin oxide (TO) is an attractive transparent conductor since tin is less expensive and scarce compared to indium. Most of the applications of architectural glass which are deposited on building windows use tin-oxide based TCOs [52]. Rutile tin oxide crystallizes in a tetragonal structure with space group P4_2_/mnm (number 136) [53]. A unit cell with lattice constants *a* = 4.7374(1) Å and *c* = 3.1864(1) Å contains two tin and four oxygen atoms. The tin cations are located at the *a* sites, and the oxygen anions are found at the *f* sites, according to Wyckoff notation [15]. Edge-sharing octahedrally coordinated tin ions form chains along the [001] direction. From the oxygen perspective, the tin atoms are located at approximately the corners of an equilateral triangle. Figure 3 shows a representation of a unit cell of rutile TO.

Samson and Fonstand [54] established that TO exhibits n-type conductivity. They observed approximately a −1/6 slope in a log (conductivity) as a function of log (*p*O_2_), similar to the behavior observed for pure In_2_O_3_. Thus, they concluded that the major defect species in SnO_2_ consists of oxygen vacancies ($V_{O}^{••}$). The non-stoichiometric decomposition and its corresponding equilibrium constant can be written as follows:

(12) $$Sn_{Sn}^{x}+2O_{O}^{x}⟺Sn_{Sn}^{x}+2V_{O}^{••}+4e^{\prime }+O_{2}$$

(13) $$K_{vac}={\left[V_{O}^{••}\right]}^{2}n^{4}P_{O_{2}}$$

The electroneutrality condition $n=[2V_{O}^{••}]$ results in n∝PO2−16. The material can then be expressed as SnO_2−*x*_ where *x* depends on the oxidation state. As in In_2_O_3_, the hypothesized population of oxygen vacancies responsible for n-type conductivity is relatively small, so it is not possible to detect it using XRD, neutron diffraction or XRF techniques. 

EXAFS studies on undoped TO nano-powders [55,56,57] have studied the effect of particle size and disorder on short-range scales. Davis *et al.* [55] measured EXAFS and XRD on pure tin oxide nano-powders prepared by a sol-gel process. The particle size of 2 to 3 nm was determined from analyzing the peak width of the XRD peaks. The measurements at the Sn *K*-edge were conducted in transmission mode at the 9.3 station of the CLRC Daresbury Synchrotron Radiation Source. *In situ* experiments showed that the particles grew significantly at 400 °C. EXAFS spectra for higher coordination shells were reduced due to the small particle size. This observation was consistent with the results of Savin *et al.* [56] and Chadwick [57]. When the coordination numbers and bond lengths for nano-SnO_2_ were simultaneously refined, both the distances and the number of neighbors were smaller compared to bulk-SnO_2_, especially for higher coordination shells. For the first shell, the distance only shortened by 0.005 Å, and the coordination number was approximately 6. However, for second, third, and fourth shells, the coordination numbers decreased by a factor of 2, and the third Sn-O shell showed the most dramatic decrease (0.142 Å). 

Savin *et al.* [56] prepared ball-milled and sol-gel nano-samples. The particle size was determined from analysis of the XRD peaks using a conventional Cu *K*_α_ source. EXAFS measurements were performed in transmission mode at station 9.3 of the CLRC Daresbury Synchrotron Radiation Source. The particle sizes obtained ranged from 8 nm up to 86 nm (this biggest size was referred to as “bulk” sample). Compared to the bulk sample, the EXAFS signals for the smaller nano-grains were attenuated, especially for the Sn-Sn bonds near 3 and 4 Å. The attenuation was inversely related to the particle size, except for the ball-milled sample with 16 nm, which showed the weakest signal of all samples. The EXAFS data were fit using two approaches. The first one refined the radial distances and Debye-Waller factors while fixing the coordination numbers to their crystallographic values: 6, 2, 4, and 8 for the first (Sn-O), second (Sn-Sn), third (Sn-O) and fourth (Sn-Sn) coordination shells, respectively. The radial distances refined to the ideal crystallographic values: 2.05 Å, 3.19 Å, 3.64 Å, and 3.70 Å, respectively, while the Debye-Waller factors increased with decreasing particle size and also with ball-milling time. The second refinement approach refined the coordination numbers and Debye-Waller factors while fixing the radial distances. In this case, the coordination numbers refined to smaller values due to a decrease in particle size. For the 11 nm sol-gel sample, the coordination numbers were 4.8, 1.5, 2.1, and 5.4, while for the 8 nm, sol gel sample they were 5.0, 2.4, 3.2, and 4.8. For the 16 nm ball milled sample, the coordination numbers were 5.0, 2.3, 4.4, and 3.6. For this last sample, the authors concluded that, besides grain size, the possible presence of an amorphous phase and an increase in disorder were important factors leading to the reduction in the number of neighbors. This conclusion was also derived from nuclear magnetic resonance results where an increase in structural disorder was evident.

Chadwick [57] also conducted EXAFS experiments at the 9.3 station of the CLRC Daresbury Synchrotron Radiation Source. Data were collected up to *k* = 18 Å^−1^ at the Sn *K*-edge on both bulk and 3 nm nanocrystals. Compared to the bulk sample, the EXAFS for the nanocrystalline sample was attenuated, and the signal from the Sn-Sn and bonds larger than 3 Å were dramatically reduced. This observation was consistent with the work of Savin *et al.* [56] and was attributed mostly to a reduced average coordination number and not disorder. Chadwick suggested that for grains smaller than 5 nm, due to a large fraction of surface atoms, the average coordination number of tin was reduced, although crystallites and interfaces could be highly disordered and contribute as well. 

### 3.5. Sb-Doped SnO_2_

Antimony-doped tin oxide (ATO) shows improved n-type conductivity compared to pure tin oxide. Berry and Laundry [58] performed Mössbauer experiments and found that at low doping levels antimony was primarily incorporated as Sn^5+^. At increasing doping levels, more Sn^3+^ species were present but their incorporation decreased the conductivity [59]. Pyke *et al.* [60] used XRD, XRF, thermogravimetric techniques, and microscopy to study the solubility of antimony in TO at temperatures ranging from 600 °C to 1000 °C. The samples had starting compositions of Sn_1−x_Sb_x_O_2_ for 0 ≤ *x* ≤ 1. From the XRD peak widths, the average crystallite sizes increased from 4 nm to 18 nm. Equilibrium was difficult to achieve, and evaporation became problematic at higher temperatures and long annealing times. However, the results indicated that the solid solution of antimony in SnO_2_ was less than 4 atomic percent. It should be noted that in thin films and nano-powders, higher Sb concentrations can be achieved. Several investigations on the defect structure of ATO consist of XRD [61,62], neutron diffraction [63], and EXAFS measurements [62,64,65]. A summary of those reports is presented next.

Gržeta *et al.* [61] used XRD and Mössbauer spectroscopy to investigate the substitution of Sb into the rutile SnO_2_ structure using samples with different atomic doping levels: 0, 3.1(4), 6.2(6) and 11.9(5), and 14.0(7)% Sb. The concentrations in the samples were determined by atomic emission spectroscopy and particle induced x-ray emission. XRD measurements were performed using a laboratory Cu *K*_α_ source, and the data were analyzed using Rietveld refinement [28]. Analysis of the diffraction peak shapes indicated anisotropy in the nanosized grains. Mössbauer spectroscopy (^119^Sn and ^121^Sb) results determined that tin was in the 4+ oxidation state, while antimony was in both the 3+ and 5+ states. The amount of Sb^3+^ was twice as much as the Sb^5+^ species. Structural results from XRD included atomic positions, isotropic Debye-Waller factors, fractional site occupancies, and lattice parameters. Both unit cell parameters, *a* and *c*, consistently increased with Sb-doping, except for the sample with highest doping level, where the lattice cell was only slightly larger than in pure tin oxide. All samples were phase pure, except for the sample with the most antimony, where 5 wt % of the sample was Sb_2_O_3_. For this sample the total amount of antimony was 19.4(7) atomic percent only 14.0(7) at.% was actually present in the rutile structure, and the rest was present in the secondary phase. The ratio *c/a* was 0.672 for all samples. 

Compared to Sn^4+^, Sb^3+^ is 0.07 Å larger and Sb^5+^ is 0.09 Å smaller in radius, so based on ionic size alone, an increase in the lattice parameters would indicate that Sb^3+^ substitutes for tin. Gržeta *et al.* [61] allowed the structural refinement of both 3+ and 5+ ions. Fractional occupancies were reported for the phase-pure samples. For the sample with 3.1%, the occupational fraction of both Sn^4+^ and Sb^5+^ added up to 0.96, while the occupation of Sb^3+^ was 0.04(2). For the sample with 6.2%, the fractional occupation of Sn^4+^ and Sb^5+^ species refined to 0.91, and to 0.09(2) for Sb^3+^. The authors also refined the occupation of the oxygen site for these two samples. Because antimony has 51 electrons and tin has 50, in this case, the fractional occupancies obtained using a conventional Cu *K*_α_ source should be interpreted with caution, since, as mentioned in section 2.1, the oxygen signal for XRD is very weak, and elements that are next to each other in the periodic table are indistinguishable using a conventional Cu *K*_α_. Anomalous synchrotron x-ray experiments and/or neutron diffraction measurements would provide more reliable results and insight into the substitution of antimony into the tin oxide rutile structure.

**Figure 4:**
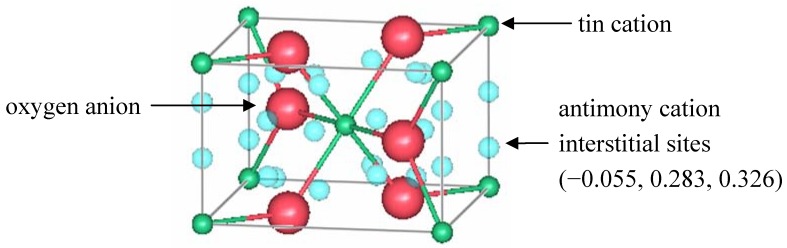
A unit cell of rutile SnO_2_ showing the location of interstitial antimony refined by Berry and Greaves [63].

Berry and Greaves [63] used neutron diffraction to investigate the defect structure of 10.6(3)% ATO with 14 nm crystallites. The measurements were performed at room temperature, using the PANDA diffractometer at ERE, Harwell. The neutron wavelength was 1.5397 Å, and the scattering lengths for tin, antimony, and oxygen were 6.1 fm, 5.6 fm, and 5.8 fm, respectively. The data were analyzed using the Rietveld method [28]. Results included atomic positions, site occupancies, isotropic Debye-Waller factors, and lattice parameters. The cell parameters were *a* = *b* = 4.7373(5) Å and *c* = 3.1816(5) Å. The best refinement scenario fixed the oxygen occupancy to 2.0 atoms/cell and refined the tin occupation in the *a* site to 0.918(7). The model also included the presence of Sb in an interstitial position (*k* site) with an occupation of 0.11(1). The interstitial antimony was initially placed at *x* = 0, *y* = *z* = 0.5, the vacant octahedral sites of the rutile structure. The refined positions were actually shifted by 1.2 Å from these octahedral sites to *x* = −0.055, *y* = 0.283, *z* = 0.326 atomic coordinates. Figure 4 shows the location of those sites in rutile. 

Berry and Greaves proposed a defect model that also includes oxygen vacancies to maintain charge balance and explain the n-type conductivity. For refinement purposes, the occupation of one species is always fixed and the rest of the sites are refined with respect to it. In the model, both cation and anion vacancies were proposed, so the relative occupancy ratios were maintained, but the absolute values were adjusted. The model then consisted of 1.90(1) O^2−^ anions, 0.871(7) Sn^4+^ cations, and 0.10(1) Sb^3+^ cations. The refined position of the antimony interstitial was in close proximity to anion vacancies. Such a Sb^3+^-Vö pair would have a net charge of 5+, which could then balance the charge of negatively charged tin vacancies.

Rockenberger *et al.* [64] measured XANES and EXAFS on samples with 9.1 and 16.7 at% antimony, with particle sizes ranging from 2 to 6 nm. The samples were prepared by a co-precipitation method, and two different antimony chlorides were used SbCl_5_ and SbCl_3_ to study their effect on the antimony oxidation state distribution. XANES experiments were performed in transmission at the Sb *L*_1_-edge at room temperature, while EXAFS measurements at the Sb *K*-edge were conducted at 5 K, at the E4 beamline of the DORIS synchrotron at HASYLAB, DESY. XANES spectra were compared to Sb_2_O_3_ (Sb^+3^) and Sb_2_O_5_ (Sb^+5^) reference materials to determine the oxidation state. The authors found that the ratio of Sb^3+^/Sb^5+^ decreased with room temperature “aging”, if the samples were dried only at 100 °C. These samples were non conductive and had a brownish, yellowish color. For the 9% Sb^3+^ sample, after 1 week at room temperature the Sb^3+^/Sb^5+^ ratio was 35/65, and it decreased to 22/78 after sitting at room temperature for 6 months. For the 17% Sb^3+^ sample, the corresponding ratios were 50/50 and 21/79; while for the 17% Sb^5+^ sample, the ratio was 9.91 (and not reported after 6 months). If the samples were dried at 500 °C, then the ratios after 1 week and 6 weeks remained the same, their n-type conductivity increased and their color changed to blue. The ratios for the 9% Sb^3+^, 17% Sb^3+^, and 17% Sb^5+^ samples were: 28/72, 23/77 and 24/76, respectively, which were all very close to 1/3. 

The EXAFS results by Rockenberger *et al.* [64] showed that the bond lengths did not change with sample composition or with drying processes. The bond lengths were: 2.00(2) Å, 3.20(2) Å and 3.71(2) Å, for the first shell (Sb-O), second shell (Sb-Sn/Sb), and third shell (Sb-Sn/Sb), respectively. These values were close to the 2.05 Å, 3.19 Å and 3.71 Å lengths in SnO_2_. The coordination numbers changed slightly with sample composition and with anneals at 100 °C and 500 °C, however most of the changes were within one standard deviation. The first coordination numbers ranged from 4.1(3) to 5.4(4). The second shell coordination numbers ranged from 0.7(4) to 1.1(5). The third shell coordination numbers ranged from 1.3(6) to 2.4(10). The authors explained that the incorporation of antimony into the rutile tin oxide lattice of their co-precipitated samples using a two-step process. During the first step at low temperatures (100 °C), the stoichiometry of the non-conductive samples was given by $Sn_{x}Sb_{y}^{III}Sb_{Z}^{V}O_{\left(2x+1.5y+2.5z\right)}$. This composition required the metal: oxygen ratio to deviate from 1:2; and their results indeed ranged from 1:2.0 to 1:2.05. Upon annealing at 500 °C, the samples became conductive and their stoichiometry was either $Sn_{x}Sb_{y-t}^{III}Sb_{Z+t}^{V}O_{2(x+y+z)}\cdot (z-y+2t)e_{CB}^{-}$ neglecting oxygen vacancies, or $(Sn,Sb)O_{2-\delta }\cdot (n+2\delta )e_{CB}^{-}$, where $e_{CB}^{-}$ represented the number of conduction band electrons, and the number of oxygen vacancies per cell was given by *δ*. Since the samples were prepared in air, such oxidizing conditions would probably not produce a substantial population of oxygen deficiencies. However, the proposed models could not be confirmed due to the typical uncertainties associated with coordination number determinations using only EXAFS results.

Geraldo *et al.* [65] also investigated the incorporation of antimony in the rutile tin oxide lattice using EXAFS and XANES. The samples were nanocrystalline powders with compositions 0%, 1.5%, 3%, 4%, 9%, and 16% Sb obtained via a xerogel process followed by drying and heat treatment at 200 °C for 30 min or at 550 °C for 1 h. XANES spectra were collected at the Sb *L*_1_-edge at station E4 of HASYLAB and compared to reference powders Sb_2_O_3_ (for Sb^3+^) and FeSbO_4_ (for Sb^5+^) to determine the oxidation state. EXAFS spectra were collected in transmission mode at the Sn *K*-edge and Sb *K*-edge on the ROMO II station at HASYLAB. XANES signals were fit using a linear combination of Sb^3+^ and Sb^5+^ dopants. The Sb^3+^/Sb^5+^ ratios of all the samples at 200 °C were smaller than the ratios after treatment at 550 °C. For the 3, 4, 9, and 16% samples, the reported ratios were: 10/90 and 0/100, 15/85 and 7/93, 55/45 and 13/87, and 70/30 and 15/85. These results differed from the 1:3 ratios reported by Rockenberger *et al.* [64] for 9% and 17% samples; and the discrepancy was attributed to a rearrangement of antimony distributions arising from different preparation routes.

EXAFS results at the Sn *K*-edge indicated that for all the low-temperature samples, the intensity of the Sn–Sn/Sb shell was reduced, probably due to the small crystallite size, which ranged from 1.1(1) nm to 1.5(1) nm. After annealing at 550 °C, the grains for most doped samples grew to 2.0(2) to 2.8(4) nm while the intensities from the Sn–Sn/Sb shell got stronger. The weakest intensities were observed for the undoped and heavily doped samples, which maintained a small crystallite size (1.4(1) nm for undoped and 1.6(2) nm for 16% Sb-doped). The bond lengths for all samples were approximately the same, within experimental uncertainty: 2.06(1) Å, 3.21(1) Å, and 3.73(1) Å for the first, second, and third shells, respectively. For all samples and temperatures the coordination number for the first shell remained constant and ranged from 5.9(2) to 6.3(1). For the second shell, after the low temperature anneal, the number of neighbors ranged from 1.2(1) to 1.4(1). After the high temperature anneal, the numbers remained the same for the undoped and heavily doped samples, but it increased to up to 1.7(1) for the other samples. The third shell exhibited more differences. The coordination numbers ranged from 2.8(2) for undoped-SnO_2_ to 3.6(2) for doped samples at low temperatures. At high temperatures, the values ranged from 3.4(2) to 5.2(3), with the undoped and heavily doped samples exhibiting the smallest numbers. The results indicated that for samples with ≤4 at.%, Sb was mostly in the Sb^5+^ oxidation state, and it favored crystallite growth resulting in stronger Sn- cation contributions that approached the behavior of bulk SnO_2_.

The environment around Sb exhibited more dramatic differences among samples and thermal treatments. The intensities for the first and second shells continuously decreased with increasing doping after both annealing treatments; however as observed before, the intensity of the cation-cation shells got stronger after heating to 550 °C. The Sb-O bond length was 1.98(1) Å for all samples. The coordination number for this shell was 5.4(5) for the 1.5% Sb sample and continuously decreased with doping to 3.9(2) for the 16% Sb sample at 200 °C. This heavily doped sample looked similar to a Sb*^III^*-grafted SnO_2_ xerogel where the Sb^3+^ species are chemically adsorbed at the surface, and the coordination number is 3.1(3). At this low temperature, the authors proposed that antimony occupied superficial sites. At 550 °C, the corresponding coordination numbers ranged from 5.7(8) to 5.3(3), which approached the value of 6 for the Sn-O bond, therefore suggesting a more homogeneous incorporation of antimony in the lattice. The authors proposed that the Sb EXAFS data could be fit using a linear combination of grafted Sb^3+^ and Sb^5+^ species. For low doping levels below 4%, the treatment at 200 °C resulted in more than 85% of the Sb^3+^ species oxidizing to Sb^5+^. As the doping increased, less than 50% of the Sb^3+^ species oxidized. At 550 °C, the Sb^5+^ species dominated and decreased with antimony content ranging from 100% to 85%.

### 3.6. F-Doped SnO_2_

Fluorine-doped tin oxide (FTO) shows improved n-type conductivity and higher mobilities compared to pure TO and ATO [1]. FTO also exhibits good chemical stability making it suitable for applications in harsh environments. At low doping levels, the incorporation of fluorine results in increased conductivity, however at higher doping levels, the conductivity decreases. Theoretical calculations by Fantini and Torriani proposed that fluorine atoms occupy substitutional oxygen sites [66] and act as a donor species providing free carriers. Due to the weak x-ray scattering of fluorine and oxygen, such data could not be used to reliably obtain fractional occupancies of these light elements in the lattice. However XRD can be used to obtain lattice parameters and to study structural changes in a semi-quantitative manner. Several structural reports [67,68,69,70] have investigated the effect of fluorine doping tin oxide.

**Figure 5:**
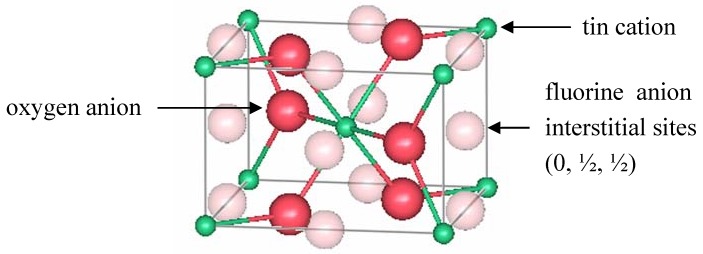
A unit cell of rutile SnO_2_ showing the location of interstitial fluorine proposed by Canestrato *et al.* [67].

Canestrato *et al.* [67] prepared undoped, doped (FTO with 0.7% F) and heavily fluorine-doped SnO_2_ (hFTO with 1.3% F) thin films which were characterized using atomic force microscopy (AFM), XRF, and XRD. The electrical and optical properties of the samples were also measured and correlated to the doping content. The resistivity of the TO sample was 32(12) × 10^−4^ Ω cm. It decreased to 6(1) × 10^−4^ Ω cm for FTO, and increased to 25(8) × 10^−4^ Ω cm for hFTO, however the carrier concentration remained approximately the same (~ 6 × 10^20^ cm^−3^) for both doping levels. Doping decreased the transmittance of the films in the IR region due to conduction electrons, affecting more the hFTO samples. XRD measurements were conducted using a Cu *K*_α_ source. Based on the intensities of several diffraction peaks, the authors concluded that F occupied both interstitial (at 0, ½, ½) and substitutional sites for both FTO and hFTO; but at higher doping levels, the occupation of the interstitial site was enhanced. Figure 5 shows the location of these interstitial sites. 

For hFTO, shifts in the Bragg angles were also consistent with the presence of interstitial atoms that caused slight distortion in the lattice. In the rutile structure, the interstitial fluorine species would act as acceptors leading to a decrease in n-type carriers. However while the conductivity decreased for hFTO, the carrier concentrations for the doped samples remained the same implying that the mobilities changed with doping. AFM showed that the morphologies for FTO and hFTO samples were comparable, ruling out the decrease in mobility due to grain boundary scattering. The authors concluded that mobility was reduced due to a combination of lattice distortion and carrier-impurity scattering, which increased with F concentration.

In a latter study, Canestrato *et al.* [68] developed a theoretical model for FTO that considered F on oxygen sites (F_*O*_), F on interstitial sites (F_*i*_) and neutralizing F_*O*_-F_*i*_ defect complexes. The authors proposed that for low doping levels, F on oxygen sites created shallow donors that increased the conductivity, while F_*i*_ might be present but inactive. However for high doping levels, F_*i*_ species were present acting as compensating acceptors. Furthermore, in heavily doped FTO, the formation of F_*O*_-F_*i*_ pairs was energetically favorable. In this complex defect, the acceptor role of F_*i*_ species resulted in a decrease in electrical conductivity. 

Suffner *et al.* [69] studied the properties of nanocrystalline FTO using XRD, TEM, x-ray photoelectron spectroscopy (XPS), and Fourier-transformed infrared spectroscopy (FTIR). XRD patterns were collected using a Cu *K*_α_ source, and the data were analyzed using the Rietveld method [28] to determine particle size and cell parameters. The average particle size of the samples was approximately 7 nm from XRD, and 5 nm from TEM measurements. The doping concentrations determined by XPS were F/Sn = 0.109 and 0.271, which translated to F/O ratios of 1:15 and 1:6. The cell volumes obtained from XRD were 71.76 Å^3^, 71.62 Å^3^, and 71.70 Å^3^ for the undoped TO, 0.109 and 0.271 FTO samples, respectively. The Sn/O ratio for pure TO was 1.80, and it decreased to 1.65 and then to 1.63 with increasing fluorine doping. These results supported the anion substitutional model, where fluorine gets incorporated on oxygen lattice sites. Because the Sn/O ratio did not change with increasing fluorine content and the volume of the cell increased, the authors proposed that at higher concentration, fluorine occupies an interstitial oxygen site. Theoretical calculations were performed to determine defect volumes of fluorine point defects and compare them with the XRD results. The calculated defects were F_*O*_ (fluorine on a lattice oxygen site), F_*i*_ (fluorine on an interstitial site) and an associate F_*O*_-F_*i*_ defect (F_*O*, *ap*_) that was proposed by Canestraro *et al.* [68]. Because the radius of fluorine is smaller compared to oxygen, it was expected that the lattice contracted with substitutional incorporation, as observed for the low doping sample. The model of isolated interstitial fluorine would lead to an overall increase in volume, while the associated defect configuration would lead to only a small cell expansion. Therefore, the authors concluded that at low doping levels, fluorine substituted for oxygen on a lattice site; and at high concentrations, the dopants went also into interstitial sites creating F_*O*_-F_*i*_ defects.

A recent study by Chinnappa *et al.* [70] also investigated the effect of doping level on the structural and electrical properties of FTO films prepared via a spray technique. The films were prepared from a low concentration (0.1 M NH_4_F, referred to as LC) and a high concentration (0.9 M NH_4_F, referred to as HC) precursor, and the atomic percentage doping levels obtained were 0, 10, 20, 30, 40, and 50. XRD, energy-dispersive x-ray analysis (EDAX), and FTIR techniques were used to characterize the polycrystalline films. From the intensity of several Bragg reflections, the authors concluded that for LC heavily doped samples, substantial F_*i*_ species were present, while for HC films, the population of F_*i*_ species was not as pronounced even for high doping levels. These results correlated well with electrical measurements. For LC films, the conductivity and carrier concentration increased with increasing doping content but after 30% F, the samples became more resistive due to decreased electron concentration. This behavior could be explained by the increasing presence of F_*i*_ acceptor species. For HC samples, the carrier concentration and resistivity did not change much with doping level, therefore it seemed like the F_*i*_ population also remained unchanged. This suggested that not only doping level, but the preparation process had an effect on the electrical properties and the fluorine doping mechanism in rutile tin oxide.

### 3.7. Wurtzite Zinc Oxide (ZnO) 

**Figure 6:**
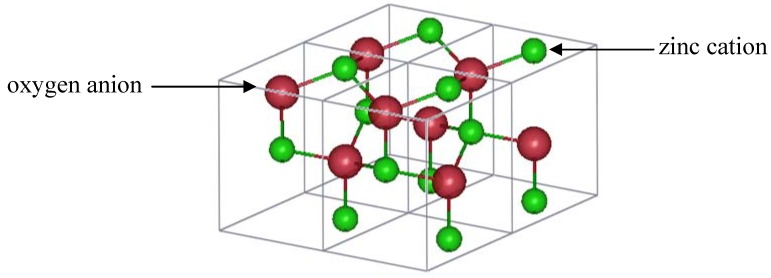
ZnO wurtzite structure (four unit cells are shown).

Zinc oxide has attracted much attention from the scientific community for several reasons: zinc is non-toxic, abundant, and less expensive than indium, making it an attractive substitute to indium-based TCOs. In the last ten years, recent efforts have also focused on achieving significant p-type doping [2,71,72,73,74,75], which is advantageous for commercial applications. Furthermore, ZnO exhibits green luminescence, which makes it even more desirable for industrial uses. Zinc oxide crystallizes in the hexagonal wurtzite structure with space group P6_3_mc (number 186) [76,77]. As shown in Figure 6, one unit cell has a total of two zinc and two oxygen ions. Zinc is tetrahedrally coordinated to oxygen. Three Zn-O bond lengths are 1.98 Å and the fourth length is 1.99 Å. Oxygen anions also exhibit tetrahedral coordination to zinc cations. The lattice parameters of pure ZnO at room temperature are *a* = *b* = 3.2249 Å, and *c* = 5.2066 Å, resulting in a *c/a* ratio of 1.602. The atomic lattice has enough space to accommodate interstitial defects.

The defect structure of ZnO remains a controversial topic due to experimental difficulties in providing conclusive support for different proposed defect models. Theoretical calculations have aimed at explaining the n-type conductivity of undoped ZnO using various defect models [5,6,7,8,9,78]. Zinc interstitials (Zn*_i_*), oxygen interstitials (O*_i_*), oxygen vacancies (V*_O_*), zinc vacancies (V*_Zn_*), zinc antisites (Zn*_O_*), oxygen antisites (O*_Zn_*), hydrogen impurities, and/or defect complexes (such as V*_O_*-Zn*_i_*) have all been considered as possible defects. Formation energies for different charged, neutral, isolated, and complex defects and their location in the energy bands have been reported. 

Kohan *et al.* [9] suggested that both zinc and oxygen vacancies were the most abundant native defects whose populations depend on the zinc partial pressure, and that the green luminescence was due to transitions between electrons in the conduction band and zinc vacancy levels and the presence of oxygen vacancies. On the other hand, Lany and Zunger [5] predicted that V_O_ was the major defect resulting in non-stoichiometry in equilibrium-grown ZnO, while Zn*_i_* species had large formation energies. Van de Walle proposed that none of the native defects were responsible for the conductivity, but that the dominant defect consisted of H^+^ impurities usually incorporated unintentionally during synthesis on interstitial sites [6]. Janotti and Van de Walle [78] concluded that zinc vacancies were deep acceptors with low formation energies that can act as compensating defects in n-type ZnO. Furthermore, their calculations indicated that oxygen vacancies were deep donors with high formation energies but donor impurities such as substitutional hydrogen (on an oxygen site) were most likely responsible for the n-type conductivity. Experimental reports [79,80,81,82] have pursued the idea of H as a cause of n-type conductivity in both intentionally hydrogen “doped” specimens and in samples without an external hydrogen source.

Lee *et al.* [83] proposed and calculated the formation energies of different types of oxygen interstitials. Erhart *et al.* [7,8] further investigated these defects that were predicted to exist under oxygen-rich conditions in the lower half of the band gap. The defects were referred to as octahedral interstitial (O*_i_*_, *oct*_), and oxygen dumbbell interstitials (or split interstitial defects). The dumbbell configurations can be regular (O*_i_*_, *db*_) or rotated (O*_i_*_, *rot-db*_). The two oxygen atoms in the regular dumbbell defect had an oxidation state of −1 and occupied an oxygen lattice site. The defect was amphoteric acting as a donor and acceptor located at a deep state. Erhart *et al.* [7] proposed that regular dumbbell configuration may compensate for p-type doping in oxidizing conditions while the rotated dumbbell defect may compensate in n-type ZnO. Also under oxygen-rich conditions, but in the upper half of the band gap, zinc vacancies were predicted to be energetically stable. While under zinc-rich conditions, oxygen vacancies would be favored.

Ischenko *et al.* [84] prepared nanoparticles and studied their defects using XRD, EXAFS, FTIR, electron paramagnetic resonance (EPR), photoluminescence (PL), and magic-angle spinning nuclear magnetic resonance (MAS-NMR). The samples were prepared from an organometallic precursor. EXAFS measurements were conducted at the Hasylab E4 station at the Zn *K* edge in transmission mode. XRD measurements were performed using a conventional Cu *K_α_* source and a position-sensitive detector. The diffraction data were analyzed to obtain lattice parameters and anisotropic particle size and strain. At 150 °C, XRD and EXAFS measurements indicated that long-range order was absent due to the presence of nanosized crystallites. As the temperature increased up to 450 °C, the particles grew to 25 nm and the XRD peaks sharpened. Coordination numbers and bond lengths were not reported but the EXAFS spectra from low thermolysis temperatures were significantly attenuated compared to bulk samples, indicating the effect of defects. As the temperature was increased, the EXAFS approached the bulk behavior. The *c* lattice parameter decreased almost linearly with annealing temperature, while the *a* lattice parameter exhibited a non-linear increase. This effect was consistent with the anisotropy in both the grain morphology and microstrain.

Ischenko *et al.* [84] also utilized EPR spectroscopy to study the presence of impurities and oxygen vacancies in the samples. Two types of paramagnetic signals were observed. A low-field signal was attributed to an unpaired electron trapped on an oxygen vacancy site (*V_O_*^+^), while the nature high-field signal was less clear. The authors proposed that the origin of the high-field signal was due to one electron weakly bound to ionized impurity atoms (e.g., C, Al, Ga, In). The authors also reported that under equilibrium conditions the predicted concentration of oxygen vacancies at 800 °C was extremely small, on the order of 10^−5^ mol% (ZnO_0.99993_). By increasing the thermolysis rates at which samples were prepared, both signals became stronger indicating the presence of more defects under non-equilibrium conditions. Annealing at up to 800 °C resulted in a reduction of defect population. An interesting observation was that when ZnO was annealed in Zn vapor at 850 °C or in vacuum, the signal attributed to impurities was present but the signal due to oxygen vacancies was absent. The authors suggested that the effect of high Zn-rich conditions was the creation of more Zn*_i_* defects instead of oxygen vacancies, contrary to theoretical predictions. Vlasenko and Watkins [85] created defects in ZnO by electron irradiation, and using optical detection of electron paramagnetic resonance (ODEPR) they detected the presence of Zn*_i_*^+^ in ZnO as an isolated species and as a Frenkel pair with *V_Zn_^−^*. These zinc defects were introduced at 4.2 K and were highly mobile with temperature annealing; however they disappeared by 200 K and were not stable at room temperature. This ODEPR study also found that oxygen vacancies were deep level defects [85,86]. The photoluminescence of the different samples prepared by Ischenko *et al.* [84] depended on sample preparation conditions, and no direct correlation was found between green luminescence and the concentration of oxygen vacancies as proposed by Kohan *et al.* [9].

EXAFS and XANES studies on thin films and nano-powders of ZnO were investigated by several research groups [87,88,89,90]. Tran *et al.* [87] studied nanocrystalline thin films grown under different water conditions. EXAFS data were collected at the Australian National Beamline Facility using a fluorescence detector. The films ranged from amorphous to crystalline and highly textured at higher water pressures. For the crystalline films, the particle size was calculated by examining the XRD peaks, and ranged from 15 nm to 40 nm. XPS data showed that up to 10 at.% of carbon was present and increased with decreasing water pressures. For all films the Zn-O and Zn-Zn distances were 1.97(5) Å and 3.25 Å, respectively, which agreed with the values for bulk ZnO. In ideal ZnO, the first coordination shell has 4 neighbors and the second has 12. When allowing the coordination numbers to vary, the analysis resulted in very low coordination numbers: 1.5 to 2 for the first shell and 2 to 5 for the second shell. The authors attributed these low values to the presence of oxygen vacancies. If the Debye-Waller factors were varied while keeping the coordination numbers fixed to 4 and 12, the disorder in the films was also evident from EXAFS results.

Chiou *et al.* [88] conducted angle-dependent XANES measurements at the O *K*, Zn *L_3_* and Zn *K* edges to study nanorods. The samples were 250 nm long and 45 nm in diameter. The authors concluded that the tip surfaces of the ZnO nanorods were terminated by oxygen ions and that the nanorods were oriented along the [000ī] direction. Han *et al.* [89] also studied nanorods using orientation dependent EXAFS at the Zn *K* edge. The length of the rods along the *c* direction was 1 μm, while the diameters were 37(3) nm and 13(5) nm when grown at 500 °C and 800 °C, respectively. The *a* lattice parameters contracted by 0.04 Å while the *c* parameter expanded by 0.1 Å compared to bulk ZnO. The coordination numbers for the nanorods and the bond lengths for higher coordination shells were comparable to those of bulk. However the bond lengths for Zn-O differed from ideal ZnO. These authors concluded that the nanorods were terminated by oxygen, in agreement with the work of Chiou *et al.* [88].

Yu *et al.* [90] used angle-dependent XANES and EXAFS to study the local structure of ZnO thin films. XRD measurements were performed at the 10B X RS KIST-PAL beamline while EXAFS experiments were carried out at the 3 C1 beamline of the Pohang Light Source in Korea. The films were epitaxial, and strain decreased with increasing film thickness, up to 800 Å. The coordination numbers were fixed to ideal ZnO while bond lengths and Debye-Waller factors were refined. The first Zn-O coordination shells exhibited differences compared to bulk, while higher coordination shell bonds approached those of ideal ZnO. Also an increase in carrier concentration, Hall mobility and overall conductivity were measured as the films became thicker.

### 3.8. Aluminum-Doped Zinc Oxide (AZO) 

Doped ZnO exhibits improved electrical properties compared to pure ZnO. Aluminum 3+ ions result in a higher electron concentration when they substitute for zinc 4+ ions. Electron concentrations on the order of 10^20^ cm^−3^ can be obtained [73]. The binary Al_2_O_3_-ZnO phase diagram prepared by Hansson *et al.* [91] determined that the equilibrium solubility of aluminum in ZnO expressed as mol fractions were 0.002 at 1250 °C, 0.005 at 1400 °C, 0.010 mol 1550 °C and 0.089 at 1695 °C. However, for thin films and nanopowders it is possible to reach much higher doping levels due to metastable synthesis conditions. The purpose of hydrogen anneals in n-type TCOs, as mentioned before, is to increase the conductivity of the materials. In the case of ZnO, as described in the previous section, the role of hydrogen as a dopant has been proposed. In AZO, several reports have also suggested that hydrogen plays a major role in defect creation and in enhancing the n-type conductivity [92].

Lany and Zunger [5] found that substitutional Al*_Zn_* species in ZnO_0.99_Al_0.01_O were shallow donors, which were expected to create free carrier due to their low formation energy. Theoretical calculations by Bazzani *et al.* [93] evaluated substitutional, interstitial and clusters of Al defects in AZO. For low doping levels, the Al-O bonds were on the order of 1.8 Å, close to the bond length in Al_2_O_3_. This length increased with doping. Their results proposed that up to 3% Al can be occupied in substitutional Zn sites. However, for higher doping concentrations, interstitial defects having large effective masses and low electron mobilities were expected to be significant and contribute to a decrease in conductivity. Also as the Al concentration increased, the Zn atoms were forced to move into interstitial positions changing the local coordination of the Zn-O bonds and resulting in inferior optical and electrical properties.

Brehm *et al.* [94] studied the local structure of Al-, Ga-, and In-doped nanocrystalline ZnO materials. XRD measurements were performed using a Cu *K_α_* source and the data were analyzed using the Rietveld method [28] to obtain lattice parameters, microstrain, and grain size. EXAFS data were measured at the Zn, In, and Ga *K* edges in transmission mode at beamlines A1 and X1 at HASYLAB. EXAFS measurements at the Al *K* edge were not conducted but data from the Zn experiments were reported for AZO samples. EXAFS data were analyzed using a reverse Monte Carlo method that allows the simultaneous fit of spectra from two different absorbing elements [95]. The pair distribution functions obtained by this technique were analyzed to extract coordination numbers and distances. The AZO samples had 0, 2, and 5% Al with a particle size of 25.2(4), 13.3(6) nm and 11.1(4) nm, respectively. The ionic radius of Al^3+^ is smaller than that of Zn^2+^ for both tetrahedral and octahedral coordination. The *a* lattice parameter increased linearly with Al doping, while the *c* parameter decreased. Similar doping effects in particle size [96] and in the *c* parameter have been reported for nanopowders with Al-doping levels up to 25% [97]. The overall effect on doping was an increase in the volume of the lattice, a decrease in the *c*/*a* ratio, and an increase in microstrain probably due to the shortening of the Zn-O bond along the *c*-axis direction. Pair distribution function data for AZO at the Zn edge were modeled assuming substitutional Al and resulted in a splitting of the first Al peak. The authors interpreted this result as an indication that Al did not substitute into the Zn sites but rather occupied octahedral interstitial sites. 

## 4. Conclusions 

This manuscript has reviewed the defect structures of several indium-, tin-, and zinc-based n-type TCOs. Isolated and complex defects in these materials play an important role in the optical and electrical behavior of these materials. Understanding their defect mechanisms provides insight on the effect of doping, grain size, synthesis and treatment processes allowing an optimization of preparation techniques to meet the demands of commercial applications. X-ray and neutron scattering are powerful techniques that provide information about short- and long-range ordering of atoms, impurity determination and quantification, the location and population of most structural defects, and their effect on the microstructure of crystalline and amorphous TCOs. More experimental studies using these characterization techniques in combination with the measurement of electrical and optical properties and a close collaboration with theoreticians are needed to further elucidate the defect mechanisms of current and future TCOs.
